# Mrs. Margret Munro Rochester

**Published:** 1890-02

**Authors:** 


### Obituary.

---

Mrs. Margret Munro Rochester, relict of the late Dr. Thomas F. Rochester, died at her residence in this city, on Monday, January 6, 1890, aged sixty-six years. Mrs. Rochester was the daughter of the late Bishop De Lancey, and has resided in Buffalo since 1853, when Dr. Rochester came here from New York to engage in the practice of his profession, and to occupy the teaching chair in Buffalo Medical College just then vacated by Dr. Austin Flint, the founder of this Journal. She was for many years Chairman of the Board of Auxiliary Managers of Buffalo General Hospital, and in many ways has lent her influence to the promotion of church and charitable interests in a substantial manner. A large circle of admiring and loving friends unite in sorrowful sympathy with the afflicted family.

